# Reflective questioning to guide socially just global health reform: a narrative review and expert elicitation

**DOI:** 10.1186/s12939-023-02083-2

**Published:** 2024-01-05

**Authors:** Sarah Lebu, Lena Musoka, Jay P. Graham

**Affiliations:** 1 School of Public Health, University of California Berkeley, 2121, Berkeley Way, Berkeley, CA 94704 USA; 2 University of North Carolina, Gillings School of Public Health, Chapel Hill, NC USA; 3 Georgetown University, McDonough School of Business, Washington, DC USA

**Keywords:** Global health, Racism, Colonialism, Decolonizing, Social Justice, International development

## Abstract

Recent research has highlighted the impacts of colonialism and racism in global health, yet few studies have presented concrete steps toward addressing the problems. We conducted a narrative review to identify published evidence that documented guiding frameworks for enhancing equity and inclusion in global health research and practice (GHRP). Based on this narrative review, we developed a questionnaire with a series of reflection questions related on commonly reported challenges related to diversity, inclusion, equity, and power imbalances. To reach consensus on a set of priority questions relevant to each theme, the questionnaire was sent to a sample of 18 global health experts virtually and two rounds of iterations were conducted. Results identified eight thematic areas and 19 reflective questions that can assist global health researchers and practitioners striving to implement socially just global health reforms. Key elements identified for improving GHRP include: (1) aiming to understand the historical context and power dynamics within the areas touched by the program; (2) promoting and mobilizing local stakeholders and leadership and ensuring measures for their participation in decision-making; (3) ensuring that knowledge products are co-produced and more equitably accessible; (4) establishing a more holistic feedback and accountability system to understand needed reforms based on local perspectives; and (5) applying systems thinking to addressing challenges and encouraging approaches that can be sustained long-term. GHRP professionals should reflect more deeply on how their goals align with those of their in-country collaborators. The consistent application of reflective processes has the potential to shift GHRP towards increased equity.

**Supplementary Information:**

The online version contains supplementary material available at 10.1186/s12939-023-02083-2.

## Introduction

The large differences in health that we observe across the globe did not take place over a short period of time but rather evolved from historical events. These inequities have recently been made even more blatant in the COVID-19 pandemic [1–5]. One of the most important influences on the disparities we see in human health has been colonialism, which was followed by neocolonialism, shifting the landscape from direct colonial power to indirect economic control by various actors in affluent countries [6, 7]. Research suggests that there was much less inequality and smaller differences between developed and developing countries before colonialism [8, 9]. Today, however, the differences in the relative burden of disease between low-middle-income and high-income countries can be an order of magnitude apart. Researchers are increasingly recognizing the effect of colonialism and the continuing systems of domination and exclusion on health and well-being [10, 11]. As an example of this increased recognition, there was a 17-fold increase in articles identified from a PubMed search of “colonialism” AND “global health” in 2020 vs. 2010. Much of the literature identified the problem, however, fewer articles set out to establish guiding principles that can potentially improve equity in GHRP [12–17].

Prior to the use of “global health”, the term used for decades was “international health.” Research suggests that international health was often guided by unequal relationships where organizations and individuals from high-income countries unilaterally set out to “help” lower-income countries deal with the public health problems defined by the higher-income countries [18–20]. Global health is generally framed as being collaborative and transnational and promoting health for all, however, it often entails partnerships between organizations in countries across the wealth spectrum (low-, middle- and high-income). Many of the countries involved in global health programs and research were previously colonized, and resource extraction in these countries – both natural and human resources – by colonizing countries was the primary objective [21]. Little attention has been paid to these historical colonial relationships and the ongoing impacts they have on global health research and practice (GHRP) [22]. We apply the same definition of “decolonizing global health” as Eichbaum et al., that is, “…removing colonial structures to include decolonization of the mind that made the colonizer feel superior and the colonized inferior by enforcing structural drivers of discrimination and barriers to self-determination” [22].

There are systems in place that diminish local autonomy and decolonized leadership in GHRP. One example of remaining systemic problems is the lack of full buy-in to the Paris Declaration on Aid Effectiveness, signed by only half of the countries in the world [9]. The Paris Declaration recognized that aid should be much more effective and addressed five core pillars to improving aid effectiveness: (1) ownership by the recipient country; (2) alignment of donor governments with the recipient country’s goals, (3) harmonization with the recipient country’s systems, (4) management of resources and decision-making focusing on results and (5) mutual accountability to improve transparency. One central aim was to begin allowing aid to be coordinated by and directly given to the recipient government in line with its priorities. This work is increasingly taking place as countries take more ownership of aid activities and establish their own national goals and systems for using aid per their policies and priorities. Research on actualizing the Paris Declaration, however, has been mixed; it appears that donor governments have failed to design their results systems to support partner country priorities [23]. This highlights the continuing unequal power relationships between donors and partners [4].

There has been an effort to mitigate the effects of colonial legacies such as inequity and power imbalance in GHRP. Notable examples include The Global Diet and Activity research Network (GDAR Network), a global public health partnership to address upstream risk factors for neglected tropical diseases in urban low and middle-income contexts. In this partnership, the GDAR Network involved non-academic partners as core resources for co-creating research materials and the research agenda, with an emphasis that ‘*No one partner dominates, and no one partner has the breadth or depth of expertise that exists across the network’* [24]. In addition, the network independently evaluated its impact based on its collective network functioning and sharing knowledge across sites, and ability to inform national and regional policy [24]. ZikaPLAN, a transnational research consortium between the European Commission and multiple Latin American countries addressing Zika, has set up an open-access community of practice to accelerate and streamline research [25]. The platform contains an entomological data repository, resources section, and networking features to create a community of practice among researchers and workers involved in vector control. Another successful national-scale adoption of equity-centered principles for GHRP is the Canadian Coalition of Global Health Research Principles for Global Health Research, which can serve as a broad-reaching and aspirational framework to guide how equity considerations are integrated into everyday research, knowledge translation, and practice [26].

Despite these efforts, there is a growing unease about whether guiding principles are effectively aligned with successful practices. Many studies that advocate for inclusive and equitable GHRP are often hampered by two major limitations. The first problem is that many of them are presented as case studies with illuminating examples - which are too narrowly focused and have limited applicability. A complex concept like decolonizing global health involves multiple actors who may frame the problem differently and see potential solutions differently based on their positionality [2]. Second, most studies are overly prescriptive, leaving little room for dynamic and creative thinking about how researchers and practitioners can tailor their programs to enhance equity and inclusion in their contexts. Prescriptive guidelines have the tendency to be overly linear, and they may even be met with significant resistance if there is no opportunity for engagement, reflection, and learning.

The objectives of this study are to (1) to examine existing frameworks and guiding principles for stemming the negative impacts of colonialism and racism on global health, (2) provide reflexive questions that can be used to design GHRP research and programs, and (3) make recommendations for best practices to global health researchers and program implementers to address colonialism and racism. The aim is to provide researchers and practitioners with a reflective process that can be used to better understand the history of a place, build more equal and empowering partnerships, and apply systems thinking to solve global health challenges.

### Positionality statement

The authors of this article would like to describe their positionality and hence their view of the topic addressed, in accordance with best practices described in published literature [27]. The first authors of this article identify as Black, East African women with expertise in global health and international development, and with public health training received from high-income countries. The third author identifies as a white male from the United States who has worked in different sectors of GHRP: (1) community-based organization, (2) an international aid agency (U.S. Agency for International Development), and (3) as a global health researcher. All authors worked as a team and brought together their experiences, expertise, and cultural knowledge to guide this analysis. The authors acknowledge their varying positionalities and recognize that the findings in this article present an interpretation based on current evidence, our experiences, and our identities.

## Methods

We conducted a narrative review to explore problematic global health research practices and efforts aimed at learning from mistakes [28]. We searched for relevant articles on two online databases: Pubmed and Scopus. We ran the following independent search strings: (a) ((colonialism OR colonial OR coloniz* OR decoloniz*) AND ‘global health’)); (b) ((racism OR race OR racial OR discrimination) AND ‘global health’)); and (c) (Global Health Research Partnerships). Studies eligible for inclusion were (1) Studies exploring aspects of colonialism and racism in global health, (2) Studies employing any study design e.g., observational studies, reviews, and commentaries, and (3) Studies reported in English. No restrictions were placed on the search based on the date of study publication. While this article is intended to address issues relevant to GHRP, literature was also drawn from other related fields, such as education [29] and nursing [30, 31]. The original search was conducted in October 2022 and updated in December 2023 using the same criteria. Screening for titles, abstracts, and full texts was completed by three reviewers (SL, LM, and JG). Data was extracted from each article for study title, type, publication year, country, affiliation of co-authors, methods, and key summaries. We did not critique or assess the articles for quality or risk of bias in accordance with existing methodologies employed for narrative reviews [32].

The data synthesis included critiques of current GHRP practices and strategies for improving equity. Two reviewers read the articles carefully and extracted any relevant terms or descriptors used to describe the concept of decolonizing global health. In cases where the reviewers disagreed with the descriptor selected from the article, they revisited the original article until they reached a consensus. By applying inductive, open coding techniques to the descriptors of decolonizing global health, a preliminary synthesis was developed using tabulation [33]. The descriptors were then grouped into broad categories or themes.

**Fig. 1:**
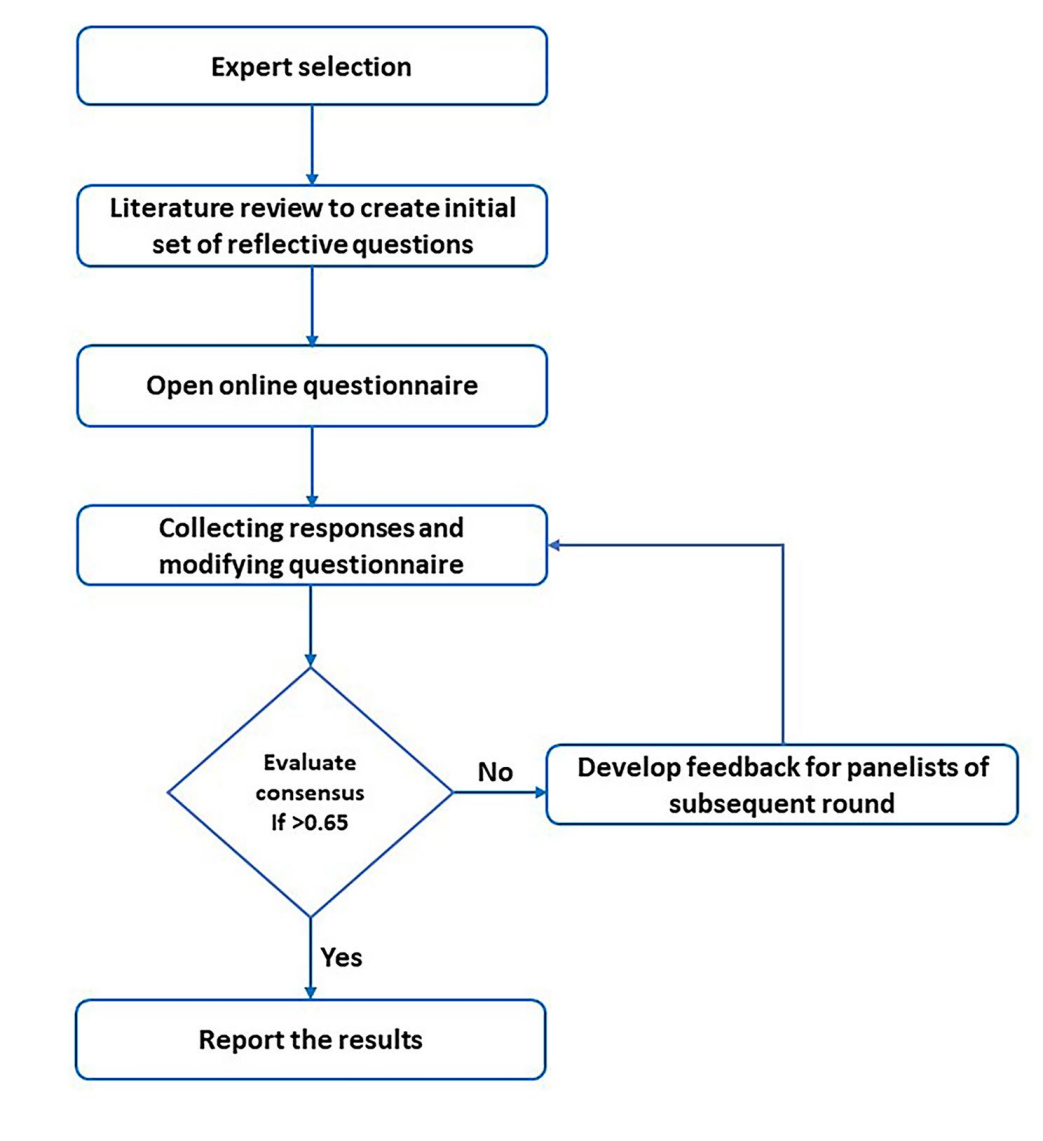
Questionnaire development and expert elicitation steps followed to determine priority questions for reflecting on socially just global health research and practice

Based on the identified themes, the authors developed draft reflective questions which were then vetted by a team of 18 GHRP professionals from low-, middle- and high-income countries, to determine which set of questions were most relevant to their circumstances. The authors identified the initial participants through their networks and expanded the sample organically through a strategic snowball approach. The sample included participants from a variety of geographical regions, age groups, genders, and years of experience. All participants agreed to participate in the process and no compensation was provided. We did not follow any formal expert elicitation protocol in this study as the knowledge area required for expert opinion did not involve quantitative content or voting. A total of two rounds of iterations were conducted for the completion of the questionnaire. Qualtrics was used to administer the survey virtually. We provide detailed information about the expert elicitation method (Fig. 1) and the survey administered in the appendix (supplementary file). Results from this process were integrated into the reflective questions presented in this article As defined in our review, reflection refers to the consideration of one’s knowledge, attitudes, and practices and their impact on others within the context of a particular social, political, and cultural setting [34].

## Results

**Fig. 2:**
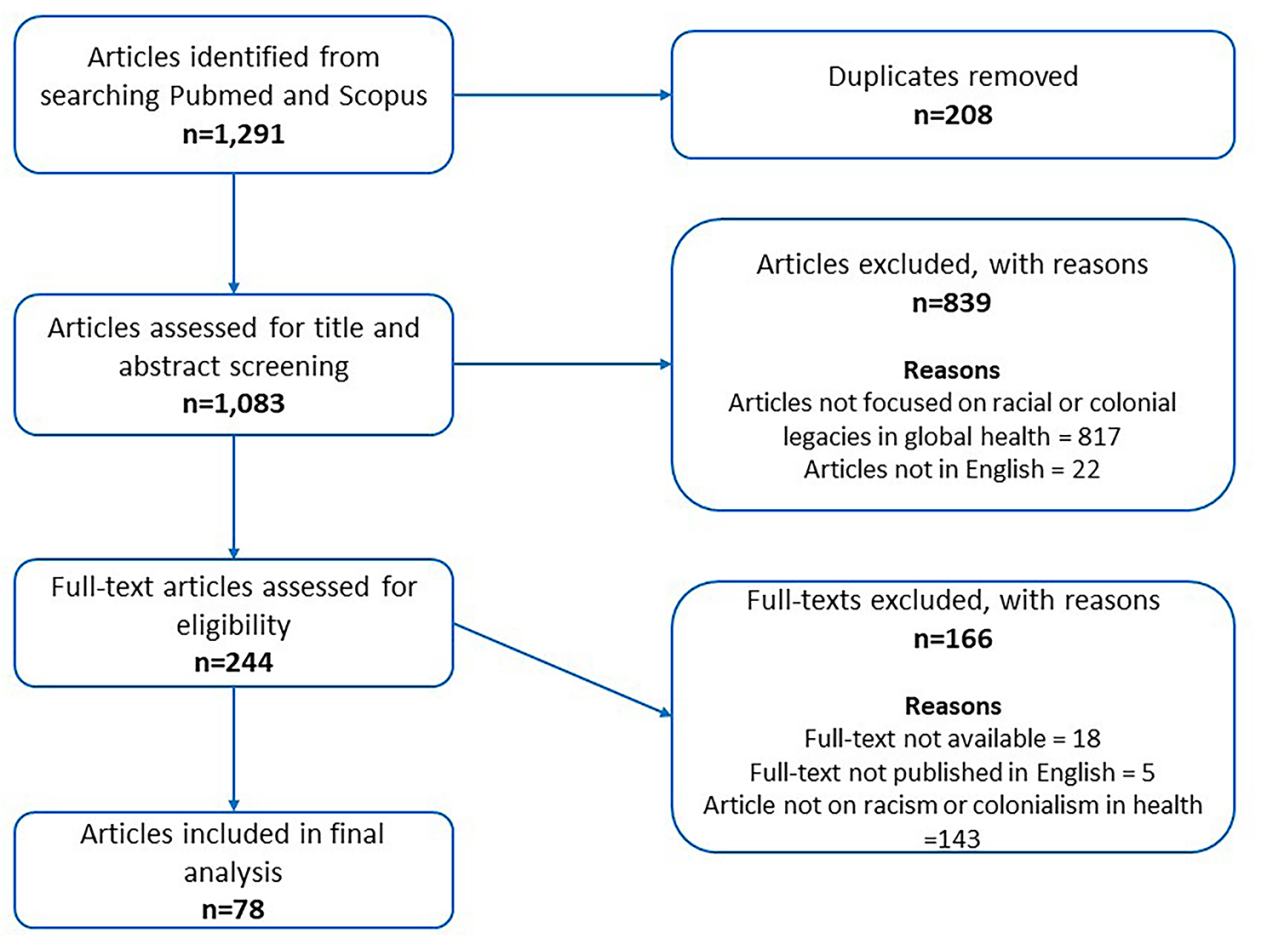
Flowchart of studies identified and included in the review

Out of the 1086 unique studies identified, 78 were included in this narrative review. A detailed flow chart of the studies identified, screened, and included in the review is provided (Fig. 2).

### Moving towards socially just global health research and practice: 8 guiding principles

We recommend GHRP use these eight principles to guide their efforts towards a more socially just global health: (1) Recognizing historical context, (2) Elevating local leadership and governance, (3) Consistently engaging local stakeholders as equal partners, (4) Strengthening capacity of local stakeholders, (5) Fostering accountability and feedback, (6) Streamlining knowledge production, access, and co-authorship, (7) Eliminating language as a structural barrier, and (8) Building systems thinking with a focus on long-term sustainability. It is acknowledged that the following principles are not exhaustive, and we recommend that they be used as a starting point for a more dynamic and sustainable reform process. We further include a list of indicators and examples of best practices to guide the implementation of each principle (Table 1).

#### Recognize the historical context

Understanding history is likely a fundamental step to reducing the detrimental impacts of colonialism and racism in GHRP [35, 36], and there is a need to more fully recognize and understand the historical events that have led to existing power imbalances and systemic inequalities [37, 38]. Recognizing the historical context stresses a need to understand colonial history and other historical events within countries, the people that GHRP aims to serve, and their perspectives, as well as the historic trauma and negative global health outcomes resulting from these events [39].

Global health is rooted in its colonial aspiration in the period of European colonialism [14]. The history of tropical medicine and global health reveals that efforts to contain diseases and plagues and improve health by reaching the ‘other’, often times had the intention of exploiting the resources of the other [40]. It is important to understand the history of GHRP programs as well as the intersectionality of racism and colonialism that affects the attitudes, practices, and policies of GHRP professionals. It is likely that understanding this history will lead to opportunities to shift individual and collective thinking to make GHRP more socially just. Acknowledging the past of global health programming, rooted in power and control, and its ongoing consequences is a first step [40]. Power imbalances remain in today’s global health system as approaches by economically advanced nations fail to promote the leadership of local communities [41]. Recognizing the historical contexts in global health approaches points to the need to tackle power imbalances and create structures that undo systemic inequities and fundamentally shift decision-making power to local communities or institutions in LMICs [31, 42].

Even the concept of using GDP as an approach to label countries is rooted in colonial practices and ignores historical processes that have led some countries to become “high-income” (e.g., the practice of enslavement or theft of natural resources). This labeling often creates a “false hierarchy among nations” [20]. A more thoughtful approach involves being more specific about why and how a setting is low-income or under-resourced and understanding the historical underpinnings [20].

#### Elevate local leadership and governance

Delivering improvements in global health will simply not be possible without addressing the fundamental element of local leadership. Leadership in institutions that set the agenda for GHRP rarely reflects the diversity of people that they intend to serve [1]. Evidence shows that more than 70% of leaders in global health funding agencies, multinational institutions, research, and non-governmental organizations are men, 80% are nationals of high-income countries and 90% were educated in high-income countries, a phenomenon described as the ‘70-80-90 glass border’ [43].

To truly level the playing field and remedy this misalignment, leadership, and decision-making authority should recognize opportunity, expertise, and achievements alongside several structural and social strata such as gender, geography, religion, economic class, race and ethnicity, age, and (dis)ability among others. There is a need to engage more women, particularly black and indigenous women in leadership positions [1, 44]. In an analysis of data from nearly all US PhD recipients and their dissertations across three decades, researchers found that demographically underrepresented students innovated at higher levels than students of the majority group, but their contributions were more often discounted and they were less likely to earn academic positions than the students in the majority group [45]. This same type of bias likely exists throughout GHRP programs.

The composition of local leadership is important to consider. For example, men and women do not always have similar decision-making power in some contexts. Other potential lines of division to be taken into consideration include class and caste; land ownership vs. landless; tenants vs. owners; lifecycle stages; marriage orders that include polygamy, where there is a female head of household or part of a joint or male-headed household, and household composition—all of which are potential drivers of health inequities [30]. These identities can intersect—for example, class and power relationships crosscut gender [46]. Thus, the role and composition of the community will vary significantly. Improving diversity and equity in local leadership can ensure many voices are effectively represented.

Notably, real, and positive impact lies in mobilizing grassroots and local capacity in a way that appropriately represents local needs and priorities. For example, GHRP commonly involves local partners after program goals and objectives have already been formulated. This undermines local stakeholders and likely misses local priorities and diminishes buy-in. Instead, programs should confront these dynamics by respecting local government and community priorities, involving and empowering local leadership, acting upon agreed health-related objectives and commitments, and operationalizing well-rounded accountability practices. Programs that respect local priorities and decisions as well as actively invite the contribution and participation of local stakeholders and strive to create balance in the face of power differences will more likely lead to sustained improvements [4, 47].

#### Consistently engage local stakeholders

GHRP professionals should seek consistent engagement with stakeholders and communities with whom they work. Though there may be a desire to improve public health for marginalized communities, some forms of community engagement in GHRP may be problematic ones guided by white saviorism, “epistemic violence”, and “unrecognized arrogance” [14, 48, 49]. These flawed world views will fail to establish consistent engagement with local stakeholders that also lift communities to a position of power to address their health issues. For instance, quick fixes that fail to acknowledge the social determinants of health or lack the involvement of local stakeholders in strategic decision-making will also fail to promote sustainable, community-led changes [50–52]. A cornerstone of GHRP should be to consistently engage and involve stakeholders and the communities throughout the process of identifying their health challenges and ways to address them [12, 53, 54]. Such involvement also ensures that programs consider communities’ priorities, local contexts, and policies, and integrate them into GHRP [49, 55].

Further challenges present themselves with global health aid and funding. Official aid agencies and development banks were not set up to be accountable to and to work for the communities they aim to serve [9]. Aid agencies are accountable to government officials and fundamentally the citizens that fund them. Multilateral development banks such as the World Bank and the Asian, African, and Inter-American Development Banks are accountable to HIC governments that sit on their boards, especially those that provide them with funding. Funding agencies have no direct accountability to LMICs although their unfulfilled needs are what justify funding agencies’ work and their funding disbursements [23].

An additional barrier that arises in GHRP is the short lead time often given in “requests for applications” and “requests for proposals” by donor institutions. These short timelines leave little time for relationship building and gaining the perspectives of local leaders and community stakeholders. Improving GHRP programs may require funds to foster stronger community relationships, and we suggest that resources may need to be made available for relationship-building efforts that can help build trust, a shared vision, and joint programmatic work plans [56].

#### Strengthen the capacity of local stakeholders

Local ownership and leadership are generally subordinate to GHRP plans and strategies directed by HICs that fund projects and this often leads to rapid shifts when priorities change. This unidirectional flow of funding has fostered a power structure and culture of supremacy that seldom places communities in the lead to either co-design a project, set priorities, drive implementation, or evaluate the impact of a program. In a research documenting contemporary experiences of partnership from the perspective of stakeholders in four sub-Saharan African research institutions, a participant reported that “*If we are really partners then we should be sitting at the table together from the beginning, all the way through the budgeting, so that it’s fair across the line”* [57]. Clearly, the ‘when’ and the ‘at what stage’ at which participants begin to get involved in research or programming are crucial.

In cases where capacity building is needed, strengthening local stakeholders to identify and prioritize their needs, and to develop and implement effective interventions is a crucial step in reducing global health inequities and improving the sustainability of programs. First, capacity building should leverage the strengths and assets of local people, government institutions, and other organizations to effectively participate in GHRP. Strengths-based approaches are likely under-utilized as tools in global health programs [58]. This can look like funding formal training and career development for local talent, supporting local organizations to build their internal capacity, and promoting collaborations in developing and publishing scientific knowledge [56, 59–61]. Organizations, such as Pre-Publication Support Service (PREPSS), are increasingly recognizing these capacity-building gaps and providing onsite training, peer-review, and copy-editing services to local researchers from LMICs who may not have the background and training to publish in journals [62]. Second, capacity building can take on the form of building community power to address systemic problems. This involves providing opportunities for local communities to have an active voice in health and development strategies. It is important to build a vibrant community that can understand, engage in and lead GHRP efforts. Initiatives such as scorecards or citizen report cards are techniques to increase local stakeholder involvement, and improve public accountability in global health services [63]. Communities have the potential to shape public health narratives and demand accountability from institutional leaders and funders [64].

#### Foster accountability and feedback

Assessment and feedback mechanisms are critical features in effective learning across many sectors; without regular feedback, GHRP professionals are unlikely to improve [8]. We suspect that systematically gathering local stakeholder feedback is lacking in global health because this transitions power to local stakeholders to constructively critique GHRP programs. Feedback remains, however, a process of information transmission dominated by GHRP professionals, not a process to let collaborators hold accountable those who may come with funding for research or programs [65]. The trend for top-down and one-way transmission of feedback will likely be a barrier to reducing power imbalances and inequities [66].

**Fig. 3:**
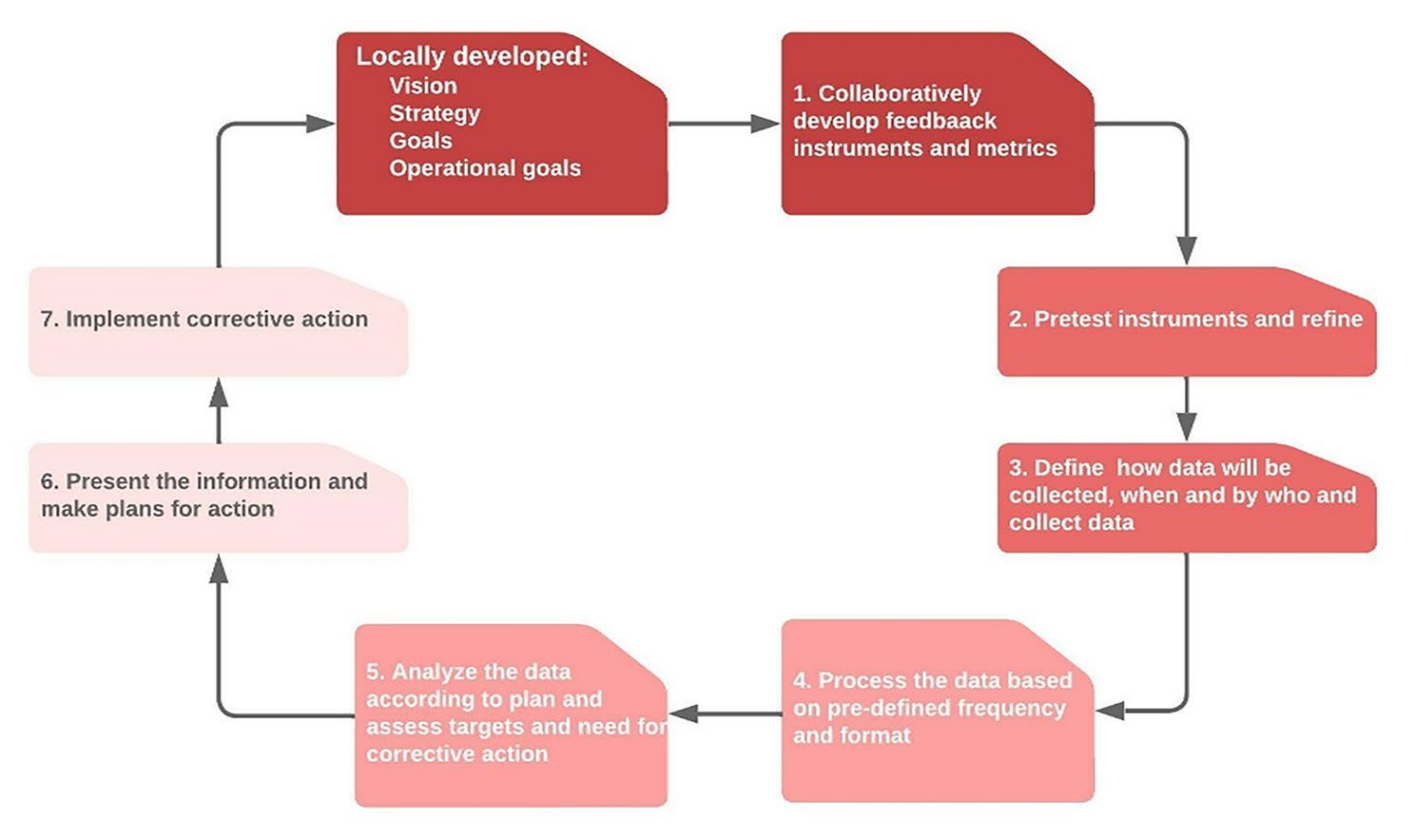
Components of a holistic feedback system where local GHRP partners review the performance of GHRP professionals

A holistic feedback system to enhance assessments by local collaborative partners and communities is needed for improving GHRP (Fig. 3) [67]. Good feedback systems address at least five key features: (1) timing - prompt and regular feedback; (2) quality - engages all stakeholders, particularly in learning what works and does not work; (3) quantity - feedback is useful in supporting efforts if it is provided often enough and on relatively small concrete activities; (4) reflection and action - use the feedback to promote reflection and set and execute action plans; and (5) communication – create more interactive and regular exchanges of information that can nurture more positive collaborations [52, 68, 69]. A successful example is the Partnership Assessment Toolkit (PAT), a practical accountability tool that enables partners to openly discuss the ethics of their partnership and to put in place mechanisms for ethical conduct [70].

#### Streamline knowledge production, access, and co-authorship

Exclusionary patterns that center Euro-Western knowledge systems have also shaped the voices that produce global health knowledge [47]. Historically, GHRP has experienced tensions between practitioners and researchers, and local communities in the resolution of global health challenges [71, 72]. Oftentimes, communities are engaged as passive participants of GRRP and not as equal partners who help shape the agenda, collect, and analyze data and implement programs [37, 47, 73, 74]. In global health research, for example, power differentials in co-authorship can result in deep structural inequities manifested in inequitable career progression and health impacts [37, 75, 76].

The growing importance of recognizing indigenous and local knowledge has been manifested through approaches such as Community-Based Participatory Research, Participatory Action Research, and more recently, citizen or street science [71, 77–81]. More effort is needed to ensure that the knowledge flow between GHRP professionals and communities is bidirectional and collaborative and that local stakeholders are both included and compensated for their contributions [82, 83]. Increasingly in global health research, shared authorship with local researchers has been described as a step to addressing power asymmetries in the production of knowledge [84], and a more flexible, collaborative model for sharing authorship with GHRP professionals from LMICs has been articulated in the literature [85, 86].

#### Eliminate language as a structural barrier

Global health research is primarily published in English; this narrows engagement and creates a structural barrier to authorship for researchers from regions where English is not their primary language [87, 88]. Relying on English only further reduces the accessibility of knowledge for non-English speaking professionals [89]. In cases where languages vary between collaborating GHRP professionals, careful consideration of which activities (e.g., monthly meetings) and products (e.g., publications and reports) will need to be translated should be collaboratively planned.

Formal translation services should be budgeted for and using translation assistance on an ad hoc and informal basis, which is common, cannot be a substitute for a more thoughtful plan for translation services. In short, financial support for document translation and simultaneous translation of meetings should always be included in global health research and practice to improve access and equity.

#### Building systems thinking with a focus on long-term sustainability

GHRP has often emphasized solutions that pay very little attention to high-level social determinants of health. For example, does GHRP consider the effects of global trade barriers or barriers to commerce that can have major effects on their programs? Systems thinking resists reductionist thinking focused on single relationships of individual components. Rather, it aims to understand something as multiple moving parts, paying attention to the complex and dynamic interactions and interdependencies of these parts.

Systems thinking is a way of looking at the individual elements of an issue, determining how those elements interconnect and affect each other (positively and negatively), and identifying points for intervention. If used effectively, systems thinking has the potential to help make more strategic decisions about how one can intervene [90]. Specific systems thinking applications that can be used to improve equity in global health include working with local partners to construct a shared vision for GHRP. In practice, local partners will likely have a much stronger understanding of the factors affecting programs, such as supply chains, price instability, and regulatory challenges that programs confront in real-world settings [91]. Building a shared understanding of the goals through collaborative model building is the first step to building trust and true partnerships. This may entail building a preliminary conceptual model by GHRP professionals, based on the relevant literature and general insights. It can’t stop there, however, and local partners must be consulted to adjust the model elements, add new elements, and more fully describe the relationships of the model elements [92]. There may likely be many differences in what stakeholders perceive are the fundamental drivers for a health problem, and collaboratively developing a causal framework is essential so that global health collaborations work toward a shared goal.

There remain significant gaps in understanding how different actors can ensure their actions are equitable and beneficial [93]. For example, how do entities partner (e.g., churches from high-income countries and churches in poor communities) effectively and equally collaborate to build infrastructure (e.g., water pumps, schools, etc.) that will be sustained into the future? How do researchers conducting systematic reviews or analyzing publicly available datasets (e.g., Demographic Health Survey and the Multiple Indicator Cluster Survey) with data from LMICs test research hypotheses while not including researchers from any of the countries where those data were collected? How does GHRP effectively transition power and leadership and reduce over-represented white males from high-income countries? How do we motivate funders to place more value on including local stakeholders in GHRP so that these individuals with specific contextual knowledge can improve intervention designs and strategies? Answering these types of questions with concrete steps, including reflective questions, will be critical for moving GHRP forward toward equity.

### Developing reflective questions for socially just global health

Based on the critiques of current GHRP practices, described above, we present a list of reflective questions that GHRP professionals can use to assess their contributions toward eliminating structural disparities and advancing more socially just practices.

The table in this section comprises three sections: Guiding principles, reflection questions, and examples of best practices and areas requiring improvement. The table should be read from left to right, starting with the guiding principles that are individually described in the section above. Each guiding principle includes a few reflective questions. These questions are based on the research articulated above. Researchers and practitioners should use these questions to think about their GHRP and gain an understanding of how should ask to see how each guiding principle can help improve equity in GHRP. The table section includes specific examples of best practices and practices that would need improvement for the corresponding guiding principle.

The purpose of this table is to launch conversations within organizations on these topics. The table and examples provided should not be viewed as prescriptive or exhaustive. Researchers and practitioners are advised to consider these questions as an opportunity to deeply interrogate their beliefs, attitudes, and practices and look for opportunities for improving equity and more fully empowering local stakeholders.

**Table 1:** Illustrative reflective questioning to guide GHRP programs and practices

		Pragmatic examples	
Guiding principles	Reflection questions	Best practices	Needs improvement
**1. Recognize the historical context**	Does your research/program work with local stakeholders to understand relevant historical events and contexts of the area?	• Local research staff leads formative/exploratory research. • Your research/program adapts lessons from previously implemented efforts.	• The program uses a one-size fits all approach for program planning, implementation, and evaluation.
	How does the program consider and potentially address past harms?	• Prioritize research on who was harmed and to what extent. • The program makes reparations for the harm done in the past.	• No research and no action on research about harm done. • Not holding perpetrators of harm accountable.
	Do local stakeholders contribute actively to agenda-setting and decision-making?	• Marginalized individuals such as women, lower castes, and persons living with a disability are included in decision-making.	• Marginalized people hold less voting power. • No recognition or compensation after marginalized people provide labor and time.
**2. Elevate local leadership**	Does the leadership team reflect the diversity of the community being served?	• Diversity by age, gender, race, caste, etc. is reflected in the composition of boards, senior leadership, and program management.	• Hiring policies are not designed for diverse candidates. • Recruitment favors candidates from privileged backgrounds (e.g., internationally recognized universities).
	Do program goals align with local priorities?	• Program goals are a direct representation of national and subnational goals. • Local stakeholders are involved in goal setting.	• Local stakeholders are informed of previously made decisions post-implementation.
**3. Engage local stakeholders as equal partners**	Do funders allow time or provide resources for partnerships to be formed between GHPRs and the communities being served?	• Constant engagement to inform local stakeholders throughout the program cycle. • Adequate time and funds allocated for local stakeholder engagement.	• Local stakeholders are engaged after the design phase of GHRP programs.
	Does your research/program identify and remove barriers to local stakeholder engagement?	• Hold meetings in accessible areas and at accessible times. • Compensate stakeholders’ time. • Hold meetings in local languages, or with interpreters available.	• Meetings held in English where most participants do not comfortably speak English. • Meetings only held online, or at times inconvenient for local stakeholders
**4. Strengthen the capacity of local stakeholders**	Has funding been allocated for capacity building?	• GHRP budget prioritizes capacity building for local stakeholders. • Provide incentives for staff professional development.	• The program only provides training on the contractual obligations of local stakeholders involved in GHRP programs.
	Does the program use a strengths-based approach to collaboratively identify and address the needs of the local stakeholders?	• Programs work with local stakeholders to assess needs in technical skills and capacity. • Hold trainings that are co-led with local stakeholders.	• Training fully led by non-local practitioners and researchers.
	Are local communities empowered to be active participants in GHRP?	• A mechanism is in place to disseminate data to stakeholders in the local language and provide opportunities to ask questions. • Communities are trained on mechanisms to hold GHRP programs accountable through feedback loops (e.g., citizen reports).	• Data shared inaccessibly, is difficult for local stakeholders to understand. • Local communities are not informed enough about research and findings to undertake community development activities.
**5. Feedback and accountability**	Has your research/program established a holistic stakeholder-centered feedback and assessment process?	• Systems for gathering feedback are in place, anonymous, and whistleblowers are protected. • Practice 360-degree feedback with local stakeholders.	• No system in place to gather stakeholder feedback. • Punitive action results from undesirable feedback.
	Has your research/program created multiple channels for local stakeholders to give prompt and regular feedback?	• Provide listening/suggestion boxes for stakeholders to give real-time feedback throughout the program.	• Accepting feedback at the beginning or end of the project only.
	How does your research/program use feedback from local stakeholders to inform implementation, promote reflection and learning?	• The program commits to redesign/restructure following local stakeholders’ feedback.	• Withhold information from local stakeholders. • Disregard local stakeholder feedback.
**6. Knowledge production, access, and co-authorship**	Are local stakeholders leading the dissemination and publication of the research (e.g., first authors of journal articles)?	• A local stakeholder is the first author of published research. • Local stakeholders are supported in developing writing and publication skills.	• Data extracted from local communities without credit or involvement of local collaborators. • Knowledge products are not shared with local communities.
	Is published research easily accessible to local stakeholders (including government officials)?	• Research results are shared in a way that local stakeholders can understand easily and make informed decisions.	• Research is published in closed-access journals whose subscriptions are unaffordable to local stakeholders.
**7. Language as a structural barrier**	Where English is not the primary language, does your program provide real-time translation of meetings and translation of meetings, research, and documents?	• A research project includes a budget to ensure that meetings and documents are translated into the local language.	• Translation of materials is done on an ad hoc basis or not at all.
**8. Systems thinking and sustainability**	Does the GHRP program work collaboratively with local stakeholders to develop a causal framework that incorporates systems thinking?	• Establish a shared vision for GHRP objectives and indicators for success. • Establish intersectoral and interdisciplinary partnerships.	• The program operates with no or limited partnerships and little understanding of the larger context.
	Does the program use a reductionist approach to implement and evaluate interventions? *Note: A reductionist approach is focused on single relationships of individual components*	• A program delivers holistic interventions that embrace contextual issues (e.g., socioeconomic, political, cultural).	• The program does not consider other existing development activities and focuses on single health outcomes.
	Does the program have a clear sustainability plan that recognizes the shortcomings of donor cycle times?	• The program develops interventions that address the short, intermediate, and long-term needs of communities.	• The program only relies on limited-term funding.

## Discussion

### Statement of principal findings

As part of this study, we identified reflective questions that can assist researchers and practitioners in moving from unfair and imbalanced practices to more socially just research and programs. The questions are intended to encourage stakeholders to work collaboratively, maintain open accountability and feedback mechanisms, and apply systems thinking approaches to achieve sustainable change. These findings make a valuable contribution to the existing body of literature, providing insights that can enhance the self-governance and leadership capabilities of local communities. Prioritizing these communities and empowering them is important, as they are given a prominent role in implementing programs aimed at improving their health and general well-being.

### Interpretation within the context of the wider literature

It is evident from these findings that global health programs and research efforts to make health care more equitable and inclusive are complex and suggest that the best way to support these efforts is to leverage the existing frameworks and tools available to them. Existing literature on diagnostic tools for identifying opportunities/processes for bias and discrimination include The BIAS Free Framework which can help identify bias along the lines of gender, race, and disability [94]. More tools that are useful for reflection and assessing alignment between program practices and the attainment of health equity have been developed, tested, and applied in different settings [95–98]. A foundational approach for equipping fairer global health practices is by focusing on academic institutions. Studies have suggested practical ways of promoting transformative praxis for learners and academic institutions of global health, including through transforming curriculum and pedagogy towards equity and decoloniality [99–105]. Producers and consumers of global health knowledge can make sense of unfair knowledge production and sharing practices using frameworks such as Epistemic Injustice Framework [106] and authorship ethics [106–108]. Studies designed to provide guidance on practicing compassionate leadership in global health without the ‘compulsion to save the world’ may also be of interest [109]. Several tools and frameworks have been developed that consider collaborations and engagement with national and subnational governments have been implemented in order to elevate country voices during the conceptualization and design of objectives and projects [22, 52, 53, 110]. Oxfam America and Save the Children have pioneered the development and application of the Local Engagement Assessment Framework (LEAF) for foreign assistance policymakers and practitioners to measure project ownership [111, 112].

We expect that those utilizing our suggested reflective questions in global health research and practice may encounter various challenges that could hinder the implementation of the recommendations. These challenges include but are not limited to the absence of political and institutional commitment, weak partnerships, limited financial resources, obstructive policies and institutional procedures, and insufficient infrastructure for disseminating information [111]. Given the restricted scope of this paper, we are unable to extensively explore each of these obstacles or provide specific approaches to tackle them. However, we encourage readers who wish to delve deeper into this subject to consult existing literature that has already been published [111].

### Limitations

Our study had several limitations. We conducted a narrative review instead of a systematic review or other comprehensive literature review design, so our findings may not be as exhaustive. Our inclusion criteria involved studies published in English due to the resources available at our disposal, thus more published reports were not represented. We acknowledge that there are approximately 7100 spoken languages globally and that English is only spoken by about 17% of the world’s population [113]. In line with other literature [113, 114], our view is that centering the English language in scientific literature (as we did in this work) is exclusionary and we recommend exploring alternative approaches to address this lingual challenge. Another limitation is that the Delphi process protocol [115] was not strictly followed, which could have caused some bias in the results even though we gained valuable insights through consulting with global experts. The initial round of questionnaire completion had a response rate of 75%, while the second round received fewer than 50% responses. Therefore, withdrawal bias may have arisen because some participants withdrew after the first round of the process. Our efforts to mitigate this were directed toward sending participants email reminders to address this issue.

## Conclusion

Progress has been made in the rhetoric of decolonizing global health, yet less has been done on the specific ways that improvements can be made. Statements made by global health donors, researchers, and international NGOs suggest that they aim to promote partner country ownership, harmonize their efforts with local stakeholders, and align with partner country priorities. At the same time, narrowly defined priorities unilaterally made by global health donors, researchers and global health organizations appear to continue.

### Electronic supplementary material

Below is the link to the electronic supplementary material.

Supplementary Material 1

## Data Availability

All data generated or analyzed during this study are included in this published article and its supplementary information files.
