# Supplementary material for: Reflective questioning to guide socially just global health reform: a narrative review and expert elicitation
Source: Int J Equity Health. 2024 Jan 5;23:3. doi: 10.1186/s12939-023-02083-2 (PMC10770991; doi:10.1186/s12939-023-02083-2)
Supplement: Supplementary file 1 — Supplementary Material 1 [file 12939_2023_2083_MOESM1_ESM.docx]

**Reflective questioning to guide socially just global health reform: a narrative review and expert elicitation**

**SUPPLEMENTARY MATERIAL**

**List of tables**

Table S1 Demographics of global health research experts who contributed towards the selection of reflective questions

Table S2 Survey administered to global health research and practice experts to elicit opinions on reflective questions

**1.0 Procedure for collecting expert opinions on reflective questions for a socially just global health**

Eighteen GHRP professionals working across twenty-five LMICs reviewed the reflective questions and provided feedback, including new questions and edits to draft questions. This information was then integrated into the reflective questions presented in this article. The demographics of the GHRP participants who contributed towards the development of the questions are shown in Table S1.

Table S1. Demographics of global health research experts who contributed towards the selection of reflective questions

|  | | N | % |
| --- | --- | --- | --- |
| Gender | |  |  |
|  | Female | 10 | 55.6 |
|  | Male | 8 | 44.4 |
| Age | |  |  |
|  | 21-29 | 3 | 16.7 |
|  | 30-39 | 6 | 33.3 |
|  | 40-49 | 4 | 22.2 |
|  | 50-59 | 4 | 22.2 |
|  | 60+ | 1 | 5.6 |
| Race* | |  |  |
|  | Black or African descent | 10 | 55.6 |
|  | Asian | 5 | 27.8 |
|  | White | 3 | 16.7 |
| Countries | |  |  |
|  | LMICs | 13 | 72.2 |
|  | HICs | 5 | 27.8 |
| Work institution | |  |  |
|  | Non-governmental/Not-for-profit | 7 | 38.9 |
|  | Academia | 3 | 33.3 |
|  | Government | 6 | 16.7 |
|  | Funders/Philanthropy | 1 | 5.6 |
|  | Private sector/Consultancy | 1 | 5.6 |
| Work experience in Global Health | |  |  |
|  | 3-5 years | 4 | 22.2 |
|  | 6-10 years | 6 | 33.3 |
|  | 11-20 years | 8 | 44.4 |

* Due to rounding, the percentage for this category may exceed 100

Table S2. Survey administered to global health research and practice experts to elicit opinions on reflective questions

My colleagues and I are in the process of developing a list of reflective questions that can be used by global health researchers and practitioners to make their work more socially just and help them to be better collaborators in the countries where they are working. We have 13 reflective questions, and we are hoping that as a global health expert, you can review and comment on the questions we’ve developed. We expect that it will take about 15 minutes for you to complete this questionnaire.

**1. Recognizing the historical context**

The following reflective questions can be used by Global Health practitioners and researchers to recognize and understand the existing power imbalances that have resulted from colonialism.

a. In your global health program or research, do local voices hold legitimate power in agenda setting and decision making?

- This sentence sounds about right
- Could use some rephrasing
  - If selected "could use some rephrasing", how would you rephrase the question?

b. Does your program recognize the history of past injustices perpetrated in the area where the program/research will take place and take steps to appropriately deal with these injustices?

- This sentence sounds about right
- Could use some rephrasing
  - If selected "could use some rephrasing", how would you rephrase the question?

c. Please write additional questions that you find important to ask in relation to recognizing historical context.

**2. Elevate local leadership and engage local stakeholders**

The following reflective questions can be used by Global Health practitioners and researchers to find ways to elevate local leadership to reflect the diversity of people they intend to serve.

a. Does the leadership team in your program reflect the diversity of the community being served?

- This sentence sounds about right
- Could use some rephrasing
  - If selected "could use some rephrasing", how would you rephrase the question?

b. Do program or research goals align with priorities of local community and stakeholders?

- This sentence sounds about right
- Could use some rephrasing
  - If selected "could use some rephrasing", how would you rephrase the question?

c. Please write additional questions that you find important to ask in relation to elevating local leadership.

**3. Strengthen capacity of local stakeholders**

The following reflective questions can be used by Global Health practitioners and researchers to find ways to empower local stakeholders to effectively participate in Global Health research and practice.

a. Does the program identify and address the training needs of local stakeholders?

- This sentence sounds about right
- Could use some rephrasing
  - If selected "could use some rephrasing", how would you rephrase the question?

b. Has funding been allocated for capacity building?

- This sentence sounds about right
- Could use some rephrasing
  - If selected "could use some rephrasing", how would you rephrase the question?

c. Please write additional questions that you find important to ask in relation to strengthening the capacity of local stakeholders.

**4. Feedback and accountability**

The following reflective questions can be used by Global Health practitioners and researchers to incorporate multidirectional feedback to reduce power imbalances and inform action.

a. Has your program created channels for local stakeholders to give prompt and regular feedback on the programs or research?

- This sentence sounds about right
- Could use some rephrasing
  - If selected "could use some rephrasing", how would you rephrase the question?

b. Does your program use feedback to promote reflection, set, and execute action plans?

- This sentence sounds about right
- Could use some rephrasing
  - If selected "could use some rephrasing", how would you rephrase the question?

c. Please write additional questions that you find important to ask in relation to incorporating feedback and accountability.

**5. Knowledge production, access, and co-authorship**

The following reflective questions can be used by Global Health practitioners and researchers to find engaging ways to involve communities as active participants and consumers of global health knowledge.

a. Are local stakeholders leading the dissemination and publication of the research (e.g., as first authors of journal articles)?

- This sentence sounds about right
- Could use some rephrasing
  - If selected "could use some rephrasing", how would you rephrase the question?

b. Is published research easily accessible to local stakeholders (e.g., government officials)?

- This sentence sounds about right
- Could use some rephrasing
  - If selected "could use some rephrasing", how would you rephrase the question?

c. Please write additional questions that you find important to ask in relation to knowledge production, access, and co-authorship.

**6. Language as a structural barrier**

The following reflective questions can be used by Global Health practitioners and researchers to remove language barriers where English is not the primary language in the area of research and practice.

a. Where English is not the primary language, does your program provide real- time translation of meetings, research, and practice documents?

- This sentence sounds about right
- Could use some rephrasing
  - If selected "could use some rephrasing", how would you rephrase the question?

b. Please write additional questions that you find important to ask in relation to language as a structural barrier.

**7. Systems thinking and sustainability**

The following reflective questions can be used by Global Health practitioners and researchers to encourage specific systems thinking strategies that can be used to construct a shared vision for global health and promote equity and sustainability.

a. Does your program deliver holistic interventions that understand contextual issues (e.g., economic, infrastructural, educational, and political)?

- This sentence sounds about right
- Could use some rephrasing
  - If selected "could use some rephrasing", how would you rephrase the question?

b. Does your program develop interventions that address both short- and long- term needs of communities?

- This sentence sounds about right
- Could use some rephrasing
  - If selected "could use some rephrasing", how would you rephrase the question?

c. Please write additional questions that you find important to ask in relation to systems thinking and sustainability.

1. What is your gender?

Female

Male

Prefer not to say

Other____________

1. Which category below includes your age?

18-20

21-29

30-39

40-49

50-59

60 or older

Prefer not to say

1. Which of these race categories do you identify with?

Asian

Black or of African descent

American Indian or Alaskan Native

Native Hawaiin or Pacific Islander

White

From multiple races

Prefer not to say

Other________

1. What country classification best describes where you are from?

Low or middle income country

High income country

Prefer not to say

1. Which institutional category does your work in global health fall into?

Academia

Government

NGO

For profit

Community-based organization

Funders/philanthropy

Other____________

1. Which country(ies) does your global health work impact directly?
2. How long have you worked in the field of global health?

0-2 years

3-5 years

6-10 years

11-20 years

More than 20 years
